# Netrin-1 and UNC5B Cooperate with Integrins to Mediate YAP-Driven Cytostasis

**DOI:** 10.1158/2767-9764.CRC-24-0101

**Published:** 2024-09-10

**Authors:** Joel D. Pearson, Katherine Huang, Louis G. Dela Pena, Benjamin Ducarouge, Patrick Mehlen, Rod Bremner

**Affiliations:** 1 Lunenfeld Tanenbaum Research Institute, Mt Sinai Hospital, Sinai Health System, Toronto, Canada.; 2 Department of Ophthalmology and Vision Science, University of Toronto, Toronto, Canada.; 3 Department of Laboratory Medicine and Pathobiology, University of Toronto, Toronto, Canada.; 4 Netris Pharma, Centre Léon Bérard 28 Rue Laennec, Lyon, France.; 5 Apoptosis, Cancer and Development Laboratory-Equipe labellisée ‘La Ligue’, LabEX DEVweCAN, Centre de Recherche en Cancérologie de Lyon, Lyon, France.; 6 Paul Albrechtsen Research Institute CancerCare Manitoba & Department of Pharmacology and Therapeutics, University of Manitoba, Winnipeg, Canada.

## Abstract

Opposite expression and pro- or anti-cancer function of YAP and its paralog TAZ/WWTR1 stratify cancers into binary YAP^on^ and YAP^off^ classes. These transcriptional coactivators are oncogenic in YAP^on^ cancers. In contrast, *YAP/TAZ* are silenced epigenetically along with their integrin and extracellular matrix adhesion target genes in neural and neuroendocrine YAP^off^ cancers (e.g., small cell lung cancer, retinoblastoma). Forced YAP/TAZ expression induces these targets, causing cytostasis in part through Integrin-αV/β5, independent of the integrin-binding RGD ligand. Other effectors of this anticancer YAP function are unknown. Here, using clustered regularly interspaced short palindromic repeats (CRISPR) screens, we link the Netrin receptor UNC5B to YAP-induced cytostasis in YAP^off^ cancers. Forced YAP expression induces UNC5B through TEAD DNA-binding partners, as either TEAD1/4-loss or a YAP mutation that disrupts TEAD-binding (S94A) blocks, whereas a TEAD-activator fusion (TEAD(DBD)-VP64) promotes UNC5B induction. Ectopic YAP expression also upregulates UNC5B relatives and their netrin ligands in YAP^off^ cancers. Netrins are considered protumorigenic, but knockout and peptide/decoy receptor blocking assays reveal that in YAP^off^ cancers, UNC5B and Netrin-1 can cooperate with integrin-αV/β5 to mediate YAP-induced cytostasis. These data pinpoint an unsuspected Netrin-1/UNC5B/integrin-αV/β5 axis as a critical effector of YAP tumor suppressor activity.

**Significance:**

Netrins are widely perceived as procancer proteins; however, we uncover an anticancer function for Netrin-1 and its receptor UNC5B.

## Introduction

Heterogeneity, plasticity, and clonal evolution drive tumor complexity, hindering successful diagnosis and treatment. Overcoming these hurdles is critical to improve cancer treatment. Identifying overarching principles of cancer biology that span tumor type and defining the molecular underpinnings of these pan-cancer rules can pinpoint broadly relevant therapeutics. We demonstrated that all cancers can be stratified into binary YAP^on^ and YAP^off^ classes with distinct genetic and therapeutic vulnerabilities, based on opposite expression and function of YAP and its paralog TAZ/WWTR1 (1).

YAP and TAZ are transcriptional coactivators that are downstream targets of the Hippo signaling pathway. In YAP^on^ cancers, YAP/TAZ are well-known oncogenes that are recruited to distal enhancers by TEAD-family DNA-binding proteins in which they cooperate with AP1 family transcription factors to induce cell cycle genes (2). In contrast, in YAP^off^ cancers, YAP and TAZ are epigenetically silenced along with less than 80 of their adhesion target genes, including integrins and extracellular matrix (ECM) proteins, such as collagens, fibronectin, and laminins, which are reactivated upon forced expression of YAP (1). Discovered through principal component analysis, we previously termed this binary-cancer-defining set as PC1^+^ genes and now refer to them as YAP^Ad^ genes (for YAP adhesion targets). YAP^off^ cancers consist of all leukemias and lymphomas, all neuroendocrine, and many neural cancers. In this context, YAP/TAZ are tumor suppressors, contrasting their oncogenic function in YAP^on^ cancers (1, 3, 4). In liquid (hematopoietic) YAP^off^ cancers, such as multiple myeloma, YAP and TAZ can induce apoptosis through various mechanisms (5–7). Alternatively, in solid neural and neuroendocrine YAP^off^ cancers, such as retinoblastoma, small cell lung cancer (SCLC), small cell neuroendocrine prostate cancer, and Merkel cell carcinoma, among others, ectopic YAP/TAZ cooperate with TEAD family proteins to induce cytostasis (1, 3). In addition, YAP silencing is also critical to permit metastasis of SCLC (8). Several YAP^off^ cancers arise from cells-of-origin that intrinsically silence *YAP/TAZ* (1), and consistent with that observation, YAP can cooperates with NOTCH and REST to antagonize neuroendocrine lineage genesis during development and repair (9).

The mechanism by which ectopic YAP/TAZ drive cytostasis in YAP^off^ cancers is incompletely understood. In retinoblastoma and SCLC, it requires YAP-induction of the Integrin-αV/β5 axis (1). Humans possess 18 α-chain and eight β-chain integrin members that can generate at least 24 different integrin α/β heterodimers (10, 11). Although some integrins are broadly expressed, others are more tissue restricted, such as β2-containing integrins expressed by leukocytes (11, 12). Integrins bind various cell surface, secreted, and ECM ligands to mediate processes such as cell–cell interactions, adhesion, cellular migration, and extravasation (10). Interaction of integrins with ECM proteins plays a central role in controlling cellular adhesion, which often involves binding of αV- or β1-containing integrins to RGD (arginine–glycine–aspartate) or related motifs in ECM proteins such as fibronectin and vitronectin (11). Interestingly, we found that, consistent with the silencing of YAP^Ad^ genes, all YAP^off^ cancers grow as non/semiadherent cultures, contrasting YAP^on^ cancers that grow as adherent cultures (1). Furthermore, forced YAP expression induced RGD-dependent adhesion of YAP^off^ cells (1). However, whereas Integrin-αV/β5 blocking antibodies attenuated YAP-induced cytostasis, they did not consistently block YAP-induced adhesion (1). Additionally, RGD peptides did not affect YAP-induced cytostasis but did disrupt YAP-induced adhesion (1). Thus, Integrin αV/β5 causes cytostasis independent of YAP-induced adhesion or Integrin-RGD interactions. However, other components that facilitate cytostasis are unknown. Here, we employ CRISPR screens to expose new effectors of YAP-induced cytostasis in YAP^off^ cancers.

## Materials and Methods

### Cell culture

Y79 (RRID:CVCL_1893) and WERI-RB1 (RRID: CVCL_1792) retinoblastoma lines were already present in the Bremner lab and were cultured in RPM1-1640 supplemented with 10% FBS. NCI-H209 (RRID: CVCL_1525) SCLC cells were obtained from Dr. Susan Cole (Queen’s University) and were cultured in RPMI-1640 with 7.5% FBS, whereas NCI-H2171 (RRID: CVCL_1536) SCLC cells were obtained from ATCC and were cultured in HITES media (DMEM:F12 supplemented with 0.005 mg/mL insulin, 0.01 mg/mL transferrin, 10 nmol/L hydrocortisone, 10 nmol/L β-estradiol, 30 nmol/L sodium selenite, 4.5 mmol/L (final concentration) L-glutamine and 5% FBS). Lenti-X 293 cells (used to generate lentiviruses) were purchased from Clontech/Takara and were cultured in DMEM with 10% FBS. Cells were maintained at 37°C and 5% CO_2_. All lines were routinely confirmed negative for mycoplasma (at least every 6 months) using the eMyco PLUS Mycoplasma PCR Detection Kit. Retinoblastoma and SCLC lines were validated by short tandem repeat analysis performed at The Centre for Applied Genomics at SickKids Hospital (Toronto, ON). Cells were maintained in culture for a maximum of 3 months before fresh aliquots were thawed and used.

### Lentivirus production

YAP and TEAD4(DBD)-VP64 vectors have been described previously (1, 13). Our YAP expression vectors are available from Addgene (Cat. #174168-174175; RRID:Addgene_174168 to RRID:Addgene_174175). Retinoblastoma lines used the PGKp series (Addgene, cat. #174172-174175), whereas SCLC lines used the EFSp series (Addgene, cat. #174168-174171). Lentiviruses were produced using Lenti-X 293 cells, as we detailed previously (13).

### CRISPR screen to identify YAP effectors

**Figure 1:**
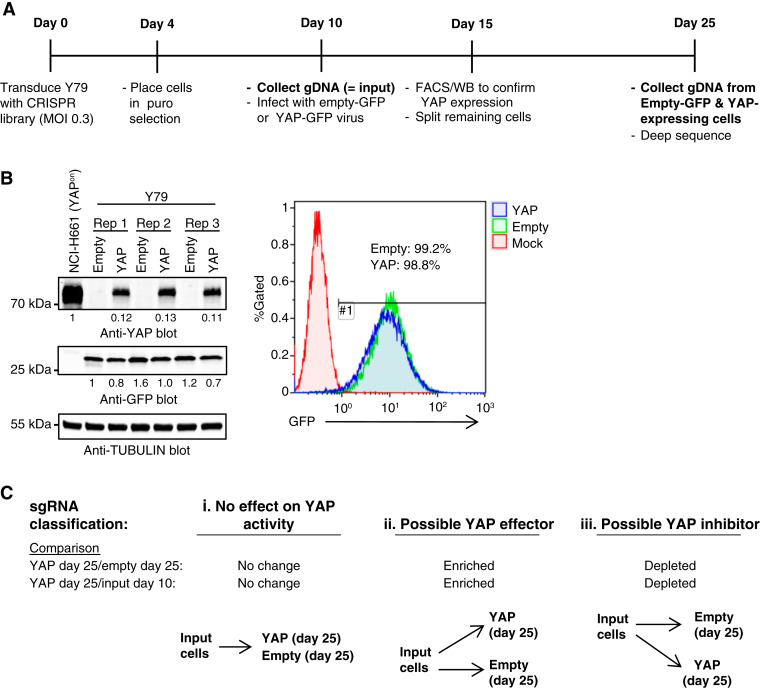
Outline of CRISPR screen to identify YAP effectors. **A,** Schematic of the CRISPR screen highlighting timepoints for viral transduction and sample collection. **B,** Western blot (left) showing expression of ectopic YAP in Y79 cells from three replicates of the CRISPR screen. YAP expression is quantified relative to YAP^on^ NCI-H661 cells. Flow cytometry plot (right) for GFP demonstrating that ∼99% of cells are transduced with the Empty or YAP expression vector. *n* = 4. **C,** Criteria used to define hits from the CRISPR screen as (i) having no effect on YAP activity; (ii) being a possible YAP effector; (iii) being a possible YAP inhibitor. YAP-expressing cells (at day 25) were compared with either Input (day 10) or Empty (day 25) cells. sgRNAs that were enriched in YAP-expressing compared with Empty or Input samples represent possible YAP effectors, whereas sgRNAs depleted in YAP-expressing cells represented possible YAP inhibitors.

The CRISPR screen was previously detailed (1) and is outlined in Fig. 1A. Briefly, a pooled CRISPR sgRNA library was constructed by cloning sgRNA sequences (4/gene plus 50 nontargeting controls) into the LentiCRISPR v2 lentiviral backbone (Addgene plasmid #52961; RRID:Addgene_52961). The sgRNA sequences were previously published (1). Then, lentivirus was generated and used to transduce Y79 cells at a multiplicity of infection of 0.3. After selection for transduced cells using puromycin, cells were collected 10 days after transduction. A portion of the cells was harvested, and genomic DNA was extracted (Qiagen DNEasy Blood and Tissue Kit) as an input sample. The remainder of the cells was transduced with either a YAP-expressing lentivirus or empty vector control. Five days later, YAP and GFP expression were confirmed using western blotting and/or flow cytometry, and then, cells were cultured for an additional 10 days (total of 15 days after YAP expression and 25 days after initial transduction with the CRISPR library). At this time, cells were harvested, and genomic DNA was isolated. The screen was performed in biological quadruplicate. Then, sgRNA sequences were PCR amplified from genomic DNA and indexed using Illumina i5 and i7 sequences. Then, these libraries were subjected to deep sequencing on an Illumina NextSeq 500 (Illumina NextSeq 500/550 Hi Output Kit v2.5 with 22 dark cycles and 26 light cycles). The resulting FASTQ files were converted to real-time base call (.bcl) files using Illumina bcl2fastq2 conversion software v2.17, and then, sequencing reads were mapped to the sgRNA library. Read counts for each sgRNA in each sample were normalized to total reads for that sample, and sgRNAs with low read counts (<20) were excluded. For each biological replicate, counts for each sgRNA in YAP-expressing cells (day 25) were compared with the Empty (day 25) and Input (day 10) samples to generate an enrichment or depletion ratio, and then, the median ratio between the replicates was calculated for each sgRNA. To prioritize possible YAP effectors, we focused on genes with ≥2 sgRNAs that were enriched ≥1.5-fold in YAP-expressing/Empty vector cells and were also enriched in YAP-expressing/Input cells. To identify possible synergistic hits (e.g., genes that increase YAP activity), we focused on genes with ≥2 sgRNAs that were depleted ≥1.5-fold in YAP-expressing/Empty vector cells, as well as in YAP-expressing/Input cells.

### Cell growth rescue experiments

To generate pooled Y79 knockout lines and appropriate controls, Y79 cells were transduced with two separate control sgRNAs (sgControl; sgControl #1—GACCGGAACGATCTCGCGTA; sgControl #2—CGCTTCCGCGGCCCGTTCAA) or two sgRNAs targeting either TEAD1 (sgTEAD1-3415—GGCCGGGAATGATTCAAACA; sgTEAD1-3418—ACATGGTGGATAGATAGCCA) or UNC5B (sgUNC5B-3692—CCAGAACGACCACGTCACAC; sgUNC5B-3693—ATACCCTAGCGATTTCGCCC) and then selected in puromycin (2 μg/mL), similar to the outline of the CRISPR screen (Fig. 1A). sgRNA sequences were cloned into the LentiCRISPR v2 vector. Approximately 10 to 12 days after the pooled lines were generated, cells were then transduced with YAP or Empty vector control (GFP only) lentiviruses so that more than 90% of cells were transduced as determined by YAP and/or GFP flow cytometry. Five days after viral transduction (peak of YAP expression), to assess YAP and GFP expression, a portion of cells were harvested for western blot and flow cytometry. The remainder of cells were plated and then counted 10 days later (15 days after YAP virus transduction). Then, the ratio of YAP-expressing to Empty vector cells was calculated for each sgRNA, and TEAD1 or UNC5B knockout lines were compared with control sgRNA expressing cells.

For netrin blocking experiments, cells were transduced with YAP-expressing or control (Empty vector) lentiviruses. The following day, cells were either left untreated or were treated with the indicated netrin blocking agent [anti-Netrin 1 (NET1-H-mAb; refs. 14, 15) or netrin trapping reagent (ectodomain UNC5-Fc; ref. 16)] at 10 or 20 μg/mL (retinoblastoma lines) or 20 μg/mL (SCLC lines). Fresh blocking agent was added to the cells every 3 to 4 days, and cells were counted 15 days after initial transduction. Integrin blocking experiments were performed similarly, except the Integrin-αV/β5 blocking antibody (Sant Cruz Biotech, sc-81632; RRID:AB_1123634) was used at 2.5 μg/mL.

### Western blotting, flow cytometry, and RT-qPCR

Our western blotting and flow cytometry protocols have been thoroughly described elsewhere (13). The following antibodies were used: YAP/TAZ (Santa Cruz Biotech, sc-101199; RRID: AB_1131430), GFP (Santa Cruz Biotech, sc-9996; RRID: AB_627695), TUBULIN (Santa Cruz Biotech, sc-32293; RRID: AB_628412), TEAD1 (BD Biosciences, 610923; RRID: AB_398238), and UNC5B (Cell Signaling Technology, 13851; RRID:AB_2798330). RNA extraction and RT-qPCR protocols have been described previously (1).

### Data availability

All data are provided in the main and supplementary figures and tables. Queries can be sent to R. Bremner.

## Results

### A CRISPR screen to identify YAP effectors

To identify key mediators of YAP tumor suppressor activity, we performed a targeted CRISPR screen in Y79 retinoblastoma cells (Fig. 1A; ref. 1). The library consisted of ∼4 sgRNAs/gene targeting ∼950 genes including YAP targets from retinoblastoma and SCLC cell lines/PC1+ genes, nontargeting sgRNAs, and controls, including sgRNAs targeting YAP, TEADs, or Hippo pathway components (Supplementary Table S1). Y79 cells were transduced with a pooled lentiviral library, and 10 days later, “input” cells were harvested. Remaining cells were then transduced with either a YAP-expressing or control (empty) vector. Both vectors coexpressed GFP to track transduced cells. After 5 days, YAP/GFP expression was confirmed by western blotting and/or flow cytometry (Fig. 1B), and then, cells were cultured an additional 10 days. At endpoint (day 25), empty vector (“Empty”) and YAP-expressing (“YAP”) cells were harvested and subjected to deep sequencing along with Input (day 10) samples. We then compared YAP-expressing cells to Empty and Input cells (Fig. 1C). First, we defined possible YAP effectors as genes with at least two sgRNAs enriched in YAP-expressing (day 25) compared with Empty (day 25) cells (Fig. 1C-ii). In addition, we prioritized genes from the latter list that also exhibited at least one sgRNA enriched in YAP-expressing (day 25) versus Input (day 10) cells (Fig. 1C-ii). Negative regulators of YAP (synergistic hits) would be depleted in YAP-expressing cells compared with Empty vector or Input cells (Fig. 1C-iii).

**Figure 2:**
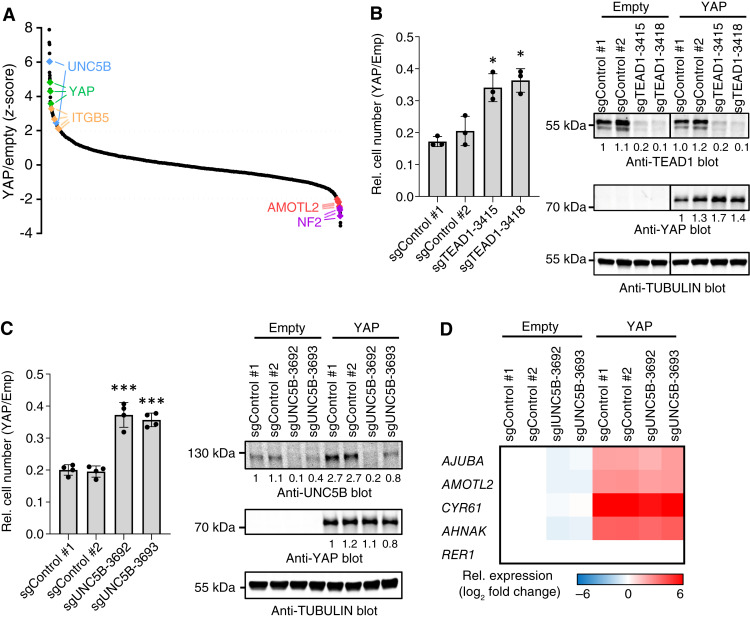
A CRISPR screen identifies UNC5B as a YAP effector. **A,***Z*-scores comparing YAP-expressing (day 25) to Empty (day 25) cells for each sgRNA in the CRISPR screen. Select genes are indicated. *n* = 4. **B,** TEAD1 knockout rescues YAP-induced cytostasis in Y79 cells (left). Western blot showing TEAD1 knockout efficiency and expression of ectopic YAP (right). *, *P* < 0.05 compared with sgControl cells, *n* = 3. **C,** UNC5B knockout rescues YAP-induced cytostasis in Y79 cells (left). Western blot showing UNC5B knockout efficiency and expression of ectopic YAP (right). ***, *P* < 0.001 compared with sgControl cells, *n* = 4. **D,** RT-qPCR for YAP target genes (*AJUBA, AMOTL2, CYR61* and *AHNAK*) or a control gene (*RER1*) in control or UNC5B knockout Y79 cells ± ectopic YAP expression. *n* = 3.

We anticipated that sgRNAs targeting YAP and TEADs would rescue YAP-induced cytostasis and would thus score as hits in the screen. Indeed, YAP sgRNAs targeting ectopic YAP rescued cell number, as did TEAD1 or TEAD4 sgRNAs despite potential redundancy with other TEAD family members (Fig. 2A; Supplementary Fig. S1A). Validation assays confirmed that TEAD1 contributed to YAP-induced cytostasis (Fig. 2B). Further validating our approach, sgRNAs targeting known YAP inhibitors exacerbated YAP-induced cytostasis. These included AMOTL2, KIRREL, and NF2, which negatively regulate YAP by activating the LATS kinases or directly sequestering YAP in the cytoplasm (Fig. 2A; Supplementary Fig. S1A; refs. 17–20). All four MORC2 sgRNAs also enhanced YAP-induced cytostasis (Supplementary Fig. S1A). We did not pursue this novel genetic interaction further here, as our focus was to identify effectors of YAP activity. As a repressor in neural cells (21), MORC2 may help silence cytostatic adhesion/ECM genes in YAP^off^ cancers. Antagonizing the effects of forced YAP expression in YAP^off^ cancer contrasts the role of MORC2 in hepatocarcinoma, a YAP^on^ cancer, in which it represses NF2 and KIBRA to promote YAP activity (22, 23).

Having identified multiple anticipated hits that validate the screen, we next focused on possible effectors of YAP tumor suppressor activity that were also induced following forced YAP expression in YAP^off^ cancers. We identified four such hits including integrin β5 (ITGB5), which we validated previously (1); the Ras family GTPase, RAB25; TGFB-induced factor homeobox 2 (TGIF2); and unc-5 netrin receptor B (UNC5B; Fig. 2A; Supplementary Fig. S1A). Follow-up experiments did not validate RAB25, but knockout of the homeodomain transcription factor TGIF2 did ameliorate YAP-mediated cytostasis (Supplementary Fig. S1B). TGIF2 was included in the screen because it is a YAP-induced target gene in SHP77 SCLC cells (1), but it was not induced by YAP in Y79 retinoblastoma cells (Supplementary Fig. S1C). RNA-Seq data from several other YAP^off^ cell lines (1) demonstrated that *TGIF2* is also not YAP-induced in these cancers. Nevertheless, whether YAP-responsive or expressed constitutively, our data indicate that this homeobox protein is an important component of YAP-induced cytostasis. Among 51 randomly chosen targets, we included in the CRISPR library that were expressed, but not YAP targets in SHP77 and Y79 cells, three of four SAP30 sgRNAs ameliorated YAP-driven cytostasis (Supplementary Table S1). SAP30 is best known as a core component of the SIN3 repressor complex, and it can also function as a coactivator (24). As our focus was on YAP-induced genes, we did not pursue this hit further, but it is noteworthy that SAP30 and TGIF2 interact (25). A genome-wide screen is needed to define the full complement of YAP cytostasis effectors in which expression is YAP-independent. Finally, validation studies with two independent sgRNAs confirmed that knocking out the YAP-inducible target UNC5B partially rescued YAP-mediated cytostasis (Fig. 2C). As we saw before for ITGB5 (1), UNC5B knockout did not affect expression of ectopic YAP or induction of YAP target genes (Fig. 2C and D), and thus, it is a downstream effector and not an upstream regulator of YAP. Hereafter, we focused on the UNC5 pathway.

### YAP regulates expression of netrins and UNC5 proteins in YAP^off^ cancers

**Figure 3:**
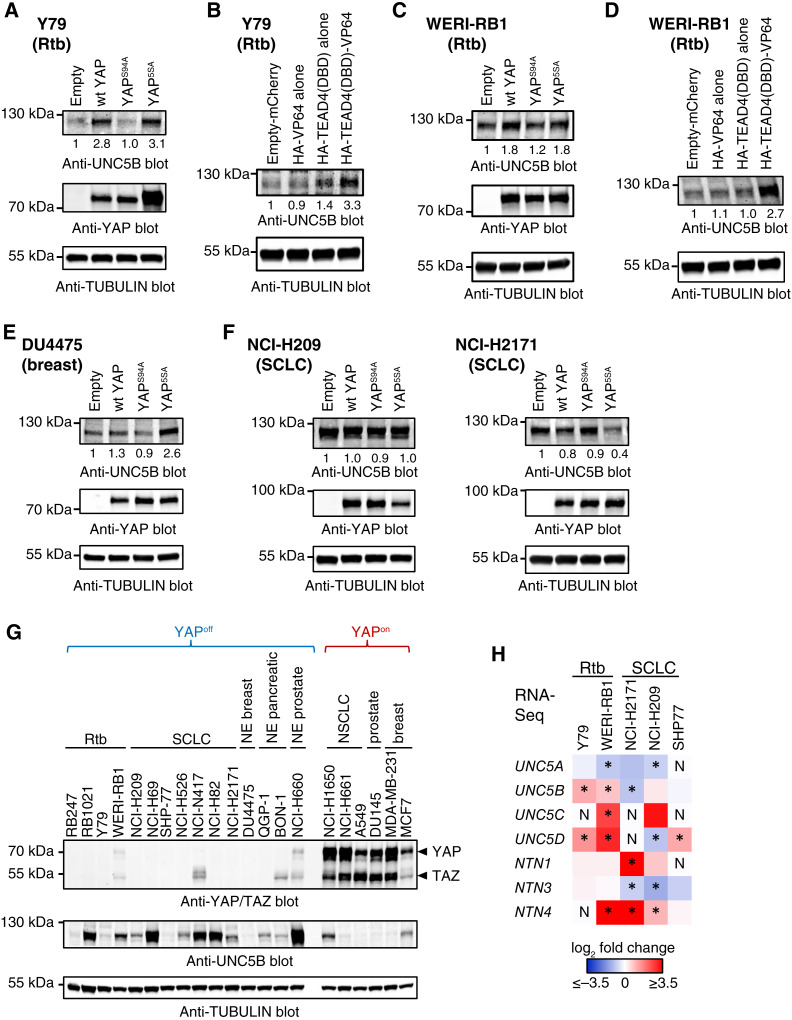
YAP induces Netrin and UNC5 family members in YAP^off^ cancers. **A–F,** UNC5B Western blots from YAP^off^ cells ectopically expressing YAP or YAP mutants (**A**, **C**, **E**, **F**) or a TEAD4 DNA-binding-domain (DBD)-VP64 fusion protein or controls (**B**, **D**). Cell lines and tumor types are indicated in each. *n* = 3. **G,** UNC5B Western blot from YAP^off^ and YAP^on^ cell lines. *n* = 2. **H,** Heatmap showing the effect of ectopic YAP on the expression of UNC5A-D or Netrins (NTN1, 2 and 4) in YAP^off^ cells. * FDR < 0.05; *N* = not detected. RNA expression levels mined from RNA-Seq data in (1).

Next, we examined the extent to which UNC5B is induced following forced YAP expression and/or constitutively expressed in YAP^off^ cancers, and whether induction is dependent on TEAD. For this, we first used a lentiviral expression system that generates levels of YAP or YAP mutants that resemble endogenous YAP levels in YAP^on^ cancers (1, 13). As predicted, wild type (wt) or constitutively active YAP (YAP^5SA^), but not the TEAD-binding mutant (YAP^S94A^), induced UNC5B in Y79 cells (Fig. 3A). The TEAD4 DNA-binding domain fused to a VP64 transcriptional activation domain (TEAD4(DBD)-VP64) recapitulates cytostasis induced by forced YAP expression in YAP^off^ cells (1). Consistent with the latter, TEAD4(DBD)-VP64 induced UNC5B in Y79 cells (Fig. 3B). YAP, YAP^5SA^, and TEAD4(DBD)-VP64, but not controls, also induced UNC5B in WERI-RB1 retinoblastoma cells (Fig. 3C and D). Moreover, YAP or YAP^5SA^, but not YAP^S94A^, induced UNC5B in DU4475 YAP^off^ breast neuroendocrine cancer cells (Fig. 3E). However, YAP did not induce UNC5B in several SCLC lines and modestly downregulated UNC5B in some cases (Fig. 3F). Whether slight downregulation of UNC5B is a direct effect of YAP or a feedback mechanism to mitigate YAP-driven cytostasis remains to be determined. Interestingly, UNC5B was already highly expressed in many SCLC (NCI-H69, NCI-N417, NCI-H82) and some other YAP^off^ cell lines (retinoblastoma/RB1021, neuroendocrine prostate/NCI-H660) relative to YAP^on^ lines (Fig. 3G), which may explain why YAP does not further increase expression in some contexts. These data suggest that intrinsically high as well as YAP-induced expression of UNC5B may facilitate YAP-driven cytostasis.

UNC5B is one of several UNC5 family members (UNC5A-D) that bind to Netrin-1 and 3 (26). Netrin-1 is a secreted protein initially described as a neuronal navigation cue, which was more recently propose to promote tumor progression in multiple human cancers (27, 28). Recent work has also implicated Netrin-3 in promoting SCLC and neuroblastoma (29). Other netrin family members such as Netrin-4 and Netrin-G1 and G2 are more divergent and do not interact with UNC5B. We asked if YAP induces other UNC5 and/or NTN members in YAP^off^ cancers. Examination of RNA-Seq data from retinoblastoma and SCLC (1) revealed up-regulation of multiple UNC5 and NTN members following forced YAP expression, including UNC5B/C/D and NTN1, but not NTN3 (Fig. 3H). Thus, YAP can induce several UNC5 receptors and their ligand NTN1 in YAP^off^ cancers.

### Blocking Netrin-UNC5B signaling rescues YAP-induced cytostasis

**Figure 4:**
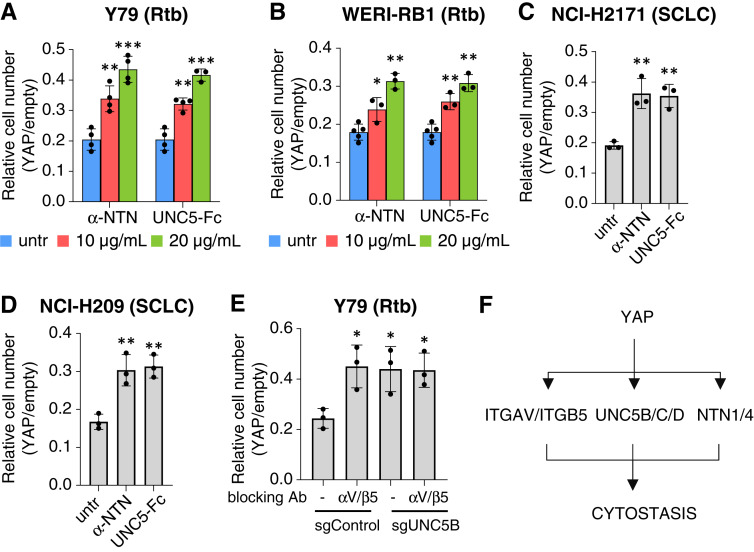
A Netrin-UNC5-ITGAV/B5 pathway mediates YAP-induced cytostasis. **A–D,** A netrin blocking antibody (α-NTN) or trapping reagent (UNC5-Fc) alleviates YAP-induced cytostasis in YAP^off^ cell lines. Cell lines and tumor types are indicated in each. Y79 cells expressed wild type YAP, whereas WERI-RB1, NCI-H209 and NCI-H2171 expressed YAP^5SA^. *, *P* < 0.05; **, *P* < 0.01; ***, *P* < 0.001 compared with untreated (untr) cells; *n* = 3–5 (**A** and **B**) or 3 (**C** and **D**). **E,** Rescue of YAP-induced cytostasis in control or UNC5B knockout Y79 cells treated with an Integrin-αV/β5 blocking antibody. *, *P* < 0.05 compared with untreated sgControl cells; *n* = 3. **F,** Summary of the mechanism of YAP-induced cytostasis in YAP^off^ cancers.

The data above reveal that forced YAP expression induces UNC5 and Netrin-1 in YAP^off^ cells, and UNC5B expression is intrinsically high in some YAP^off^ contexts. Thus, we studied the extent to which Netrin-UNC5B signaling is required for YAP-induced cytostasis across various YAP^off^ cancers. We utilized two strategies to neutralize netrin, including a blocking antibody (NET1-H-mAb; refs. 14, 15) or a Netrin-1 trapping reagent (ectodomain UNC5-Fc; ref. 16). Both strategies produced a dose-dependent rescue of YAP-induced cytostasis in Y79 cells (Fig. 4A), confirming UNC5B knockout data (Fig. 2A and C). Indeed, blocking Netrin-1 (Fig. 4A) rescued growth to a similar extent as UNC5B knockout (Fig. 2C). Blocking Netrin-1 also ameliorated YAP-induced cytostasis in WERI-RB1 retinoblastoma cells (Fig. 4B). As noted above, forced YAP expression did not increase already high UNC5B levels in SCLC lines such as NCI-H2171 and NCI-H209, but YAP-induced Netrin-1 in both contexts (Fig. 3H), and the Netrin-1 antibody and trapping agents alleviated YAP-induced cytostasis in NCI-H2171 and NCI-H209 SCLC cells (Fig. 4C and D). Netrin blockade did not affect expression of ectopic YAP or induction of YAP target genes, thus Netrin-1, like UNC5 (this work) and ITGB5 (1), act downstream of YAP (Supplementary Fig. S2). Together, these data reveal that UNC5-NTN1 signaling is a vital effector of YAP-induced cytostasis in YAP^off^ neural and neuroendocrine cancers.

Blocking the Integrin-αV/β5 dimer, either via gene deletion or with blocking antibodies, ameliorates YAP-induced cytostasis in YAP^off^ cancers (1), similar to the effects of inhibiting Netrin-1/UNC5 activity (Figs. 2C and 4A–D). Integrins and the netrin-UNC5 pathway cooperate in several settings (30–33), and thus, we asked if they similarly cooperate to mediate YAP-driven cytostasis. If these YAP effectors regulate separate pathways to induce cytostasis, targeting both would be additive or synergistic. However, if they function in the same pathway, targeting both effectors should exhibit the same effect as blocking either. Thus, we treated wild type or *UNC5B* null Y79 cells with an Integrin-αV/β5 blocking antibody. As seen before (1), inhibiting Integrin-αV/β5 in wild type Y79 cells mitigated YAP-induced cytostasis (Fig. 4E). Importantly, the magnitude of rescue with the Integrin-αV/β5 blocking antibody was identical in *UNC5B* null cells (Fig. 4E). These data suggest that netrin-UNC5 and Integrin-αV/β5 cooperate in the same pathway to mediate YAP cytostatic activity in YAP^off^ cancers (Fig. 4F).

## Discussion

A positive association between netrin and cancer progression is well-established (27). As a ligand for its cognate death receptors, over expression of netrin, either by the cancer cells or neighboring cells such as cancer associated fibroblasts, promotes survival, and also stemness (34–38). Netrin can also promote endothelial cell survival, angiogenesis, and vascular mimicry (39–43), and it supports cancer cell migration and invasion (44–48). Moreover, a recent first-in-class antinetrin therapeutic showed efficacy in a clinical trial for endometrial cancer, impeding cell survival and epithelial mesenchyme transition (49). Indeed, researchers well-established that netrin is oncogenic in various cancers, such as melanoma, pancreatic ductal carcinoma, hepatocellular carcinoma, prostate carcinoma, and endometrial cancer (44–49), and all of these are YAP^on^ cancers (1). In that context, YAP is tumorigenic, contrasting its tumor suppressor function in YAP^off^ cancers that consist of all neuroendocrine and hematopoietic cancers as well as several neural cancers (1). The potent antiproliferative effect explains why YAP and its paralog WWTR1/TAZ are silenced in YAP^off^ cancers. Here, a targeted CRISPR/Cas9 screen identified the UNC5B receptor as a key mediator of YAP-induced cytostasis in YAP^off^ cancers. Forced YAP expression induced the expression of UNC5B and the related UNC5 family receptors, UNC5C and D, as well as their ligand NTN1/Netrin-1 in YAP^off^ cancers. In contrast, mining transcriptome data from eight YAP^on^ lines with YAP or YAP/TAZ knockdown (1) revealed that YAP/TAZ do not upregulate these genes in that context (Supplementary Fig. S3), suggesting that YAP-induction of UNC5-family/NTN1 genes is YAP^off^-cancer-specific. Induction of UNC5-family and NTN1 in YAP^off^ cancers depended on YAP/TEAD-binding as YAP^S94A^, which cannot bind TEADs, failed to induce UNC5B, and a TEAD4(DBD)-VP64 fusion mimicked the effects of YAP. Whether induction of UNC5-members and/or NTN1 by YAP/TEAD is direct or indirect remains to be determined. TEAD4 ChIP-Seq data in these same YAP^off^ lines (1) did not reveal TEAD4 binding at the promoter of these genes, but YAP/TEAD primarily regulate genes via distal enhancers (1, 50–53), so it is possible that YAP/TEAD directly induce the expression of UNC5-family members and NTN1 via distant enhancers. Using either a netrin blocking antibody or a Netrin-1 trapping reagent, we demonstrated that netrin signaling is a key anticancer effector of ectopic YAP across multiple YAP^off^ contexts. In this work and a prior study (1), we focused on deducing how YAP initiates cytostasis, and thus, we specifically tested whether blocking UNC5-NTN or Integrin signaling at the onset of forced YAP expression rescues cytostasis. In future studies, it will be interesting to add blocking antibodies after cytostasis is established to deduce whether the same factors also maintain YAP-driven cytostasis. Our genetic and antibody-blocking strategies revealed that UNC5/netrin signaling cooperates with the Integrin-αV/β5 pathway to mediate the cytostatic effects of forced YAP expression in YAP^off^ cancers. It is noteworthy that forced YAP expression in YAP^off^ cancer induces multiple ECM components, including direct targets of netrin/integrin complexes such as collagens and laminins (1, 31). Thus, our work provides a coherent framework within which to understand why YAP is cytostatic in YAP^off^ cancers.

Our results linking Integrin αV/β5 (1) to the Netrin-1/UNC5 axis (this work) are consistent with functional netrin/integrin interactions observed in other settings. For example, Netrin-1 activates Integrin β1 to drive migration and metastasis of neuroblastoma and, of particular note, both proteins are in a complex together (30). Moreover, α6/β4 integrin mediates pancreatic epithelial cell adhesion to netrin-1 (31), and netrin also binds α3β1 integrin to regulate interneuron migration in the cortex (32). We envisage, therefore, that the ability of Netrin-1, UNC5, and Integrin-αV/β5 to arrest growth in YAP^off^ cancers likely involves their physical interaction, although that remains to be demonstrated formally. To our knowledge, our data provide the first example of netrin/integrin cooperation to inhibit cancer cell growth. There are indeed scant examples of netrin suppressing cancer. In another case—pancreatic ductal adenocarcinoma (PDAC), a YAP^on^ cancer (1)—netrin suppresses 3D tumor growth in xenograft models (54). In that YAP^on^ context, netrin suppresses the expression of oncogenic integrin β4 indicating netrin/integrin antagonism, which stands in stark contrast to YAP^off^ cancers in which our results reveal that Netrin-1 and integrins cooperate to inhibit proliferation.

Although YAP, TAZ, and ITGB5 are downregulated in YAP^off^ cancers (1), the UNC5 family show varying levels depending on the paralog. Our results reveal that even where UNC5B is constitutively expressed, it is required for YAP-driven cytostasis. Another hit in our screen, the homeobox protein TGIF2, was constitutively expressed in most tested YAP^off^ cancer lines, but our genetic studies suggest it is also key for YAP-mediated growth-arrest. TGIF2 promotes a variety of YAP^on^ cancers, such as ovarian and cervical cancer (55, 56). TGIF2 has been studied much less in YAP^off^ cancers, although the paralog TGIF1 suppresses acute myeloid leukemia consistent with our results in neural/neuroendocrine YAP^off^ cancers (57). TGIF2 is connected to TEAD/YAP in other contexts, as this homeobox protein can convert hepatocytes to pancreatic progenitors, which involves induction of TEAD2 (58), and TEAD/YAP induce factors that promote pancreatic development (59). Of note, constitutively expressed SAP30 was also a hit in our screen, which can interact with TGIF2 (25). Overall, whether the distinct YAP effectors that cooperate to promote cytostasis in YAP^off^ cancers are YAP-induced or constitutive is context dependent, but both groups are essential. Contexts in which constitutively expressed netrin and/or UNC5-proteins contribute to cytostasis, it is likely they are cooperating with other YAP-induced targets, such as additional UNC5 members and Integrin-β5.

Limitations exist in our study. Ectopic YAP potently arrests growth of SCLC and retinoblastoma cells *in vivo* and *in vitro* (1), and although we demonstrated an antiproliferative role for netrin, UNC5, and integrins *in vitro*, further work is required to test this effect *in vivo*. Also, we functionally assessed the anticancer netrin/UNC5/Integrin cooperation in neuroendocrine and neural cancers, and although we also observed YAP-induced expression of UNC5B in neuroendocrine breast cancer cells, additional functional analyses are required to test whether the cytostatic effect extends to this and other YAP^off^ cancers, particularly the large hematopoietic class (1). Forced YAP expression suppresses multiple neuroendocrine and neural cancers in a TEAD-dependent fashion (1, 3), although suppression of multiple myeloma by TAZ is TEAD-independent (6). In addition, our work does not show the extent to which different members of the netrin and UNC5 family are utilized to mediate context-specific antiproliferative effects of YAP. Nevertheless, this study does reveal that ectopic YAP upregulates UNC5 family members to distinct extents in YAP^off^ cancers. Although YAP-induced UNC5 and NTN mRNAs in multiple YAP^off^ cancer lines, and we confirmed that YAP or TEAD-VP64 induced UNC5B protein across multiple lines, we were unable to confirm whether other UNC5 proteins or NTN1 protein were induced in lines in which their mRNAs were upregulated. We tested a goat polyclonal antibody to UNC5C (R&D Systems, cat# AF1005), which was published to work (60), but in our hands, the antibody detected many bands, perhaps reflecting lot variation typical of polyclonal antibodies. Also, we tested a published NTN1 antibody (Abcam cat# ab126729) but did not detect protein from suspension cultures perhaps because secreted NTN1 is released in suspension cultures (which are YAP^off^ lines), and although we did detect an induced band at the appropriate size using cells adhered to polyD-lysine, numerous background bands were also found. Despite these technical hurdles in detecting all the proteins by Western, the evidence that they mediate YAP-induced cytostasis in YAP^off^ cancers is compelling. Thus, UNC5B protein-induction was detected in multiple lines, UNC5B-knockout countered YAP-induced cytostasis in Y79 retinoblastoma cells, and a Netrin blocking antibody and a Netrin trapping reagent (UNC5-Fc) also had this anticytostatic effect across multiple YAP^off^ cancer cell lines. Also, we did not interrogate rigorously the role of other netrins in YAP-mediated cytostasis. Consistent with prior work showing that Netrin-3 promotes SCLC (29), we found that Netrin-1 but not Netrin-3 was induced following forced YAP expression in this cancer. However, it is unclear how Netrin-3 would affect SCLC cells expressing YAP. We also observed Netrin-4 induction, but as this ligand does not bind the UNC5 receptor, it was not pursued further. Finally, the signals downstream of the Netrin-1/UNC5B/Integrin-αV/β5 that cause cytostasis remain to be deduced. However, of note, although netrins contain an RGD motif, interaction with integrins occurs through a distinct 25 amino acid peptide (31), providing a logical hypothesis about why YAP-induced cytostasis in YAP^off^ cancers is RGD-independent (1).

In summary, our work illuminates how YAP promotes cytostasis in YAP^off^ cancers. It also exposes a unique example of how Netrin-1 can cooperate with integrins to inhibit rather than promote cancer cell growth, underscoring the striking differences between binary YAP^off^/ YAP^on^ cancer classes.

## Supplementary Material

Supplementary Figure S1 Supplementary Figure S1: Results of CRISPR screen to identify YAP effectors

Supplementary Figure S2 Supplementary Figure S2: Blocking Netrin does not affect YAP levels or YAP target gene induction

Supplementary Figure S3 Supplementary Figure S3: Regulation of UNC5 family and NTN1 mRNA by YAP in YAP-off vs YAP-on cancers

Supplementary Table S1 Supplementary Table S1: CRISPR Screen Results
