# Supplementary material for: Netrin-1 and UNC5B Cooperate with Integrins to Mediate YAP-Driven Cytostasis
Source: Cancer Res Commun. 2024 Sep 10;4(9):2374–83. doi: 10.1158/2767-9764.CRC-24-0101 (PMC11384508; doi:10.1158/2767-9764.CRC-24-0101)
Supplement: Supplementary Figure S1 — Results of CRISPR screen to identify YAP effectors [file crc-24-0101_supplementary_figure_s1_suppsf1.pdf]

Supplementary Figure S1: Results of CRISPR screen to identify YAP effectors

A.

sgRNA	YAP/Empty (median fold enrichment)	YAP/Input (median fold enrichment)	
sgYAP1-335	1.82	1.72	Expected YAP effectors
sgYAP1-336	2.10	2.00	
sgYAP1-337	1.98	1.47	
sgYAP1-338	1.35	1.35	
sgTEAD1-3415	2.23	1.90	
sgTEAD1-3416	1.45	1.37	
sgTEAD1-3417	0.85	1.08	
sgTEAD1-3418	1.74	1.58	
sgTEAD4-3427	1.13	1.19	
sgTEAD4-3428	1.51	1.38	
sgTEAD4-3429	1.46	1.41	
sgTEAD4-3430	0.83	0.97	
sgITGB5-455	1.75	2.08	Possible effectors
sgITGB5-456	1.62	1.79	
sgITGB5-457	1.49	1.81	
sgITGB5-458	1.50	1.77	
sgTGIF2-3455	1.73	1.99	
sgTGIF2-3456	2.11	1.74	
sgTGIF2-3457	1.90	1.73	
sgTGIF2-3458	1.10	1.27	
sgUNC5B-3691	0.72	0.82	
sgUNC5B-3692	2.37	1.83	
sgUNC5B-3693	1.57	0.99	
sgUNC5B-3694	1.26	1.24	
sgRAB25-2895	1.56	1.11	
sgRAB25-2896	1.62	1.29	
sgRAB25-2897	0.93	1.02	
sgRAB25-2898	0.88	0.97	
sgAMOTL2-715	0.57	0.68	Negative regulators of YAP
sgAMOTL2-716	0.53	0.62	
sgAMOTL2-717	0.47	0.47	
sgAMOTL2-718	0.54	0.82	
sgNF2-2503	0.43	0.64	
sgNF2-2504	0.45	0.41	
sgNF2-2505	0.61	0.52	
sgNF2-2506	0.35	0.40	
sgKIRREL-2115	0.53	0.46	
sgKIRREL-2116	0.51	0.53	
sgKIRREL-2117	1.00	0.67	
sgKIRREL-2118	0.36	0.45	
sgMORC2-2351	0.26	0.30	
sgMORC2-2352	0.22	0.30	
sgMORC2-2353	0.42	0.42	
sgMORC2-2354	0.46	0.43	

B.

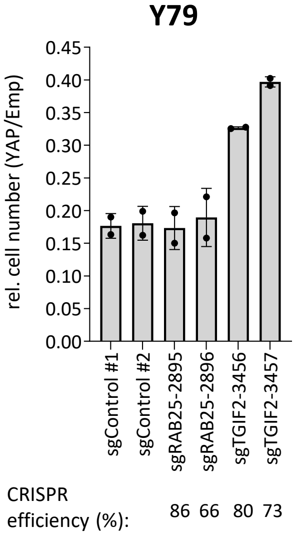

C.

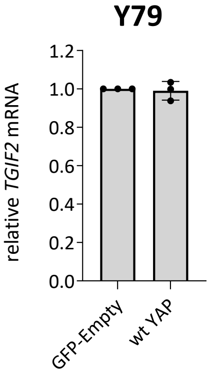

Figure S1. Results of CRISPR screen to identify YAP effectors. A. Table showing the fold difference for selected sgRNA hits between YAP (25 day) and Empty (25 day) or Input (10 day) cells. Selected hits are shown, with scoring sgRNAs indicated with shading. For a comprehensive list of hits see Table S1. **B.** TGIF2, but not RAB25 knockout alleviates YAP-induced cytostasis in Y79 cells; n = 2. CRISPR efficiency was determined by DNA sequencing followed by TIDE analysis. **C.** RT-qPCR for *TGIF2* in Empty or YAP-expressing Y79 cells. n = 3.
