# Supplementary material for: Netrin-1 and UNC5B Cooperate with Integrins to Mediate YAP-Driven Cytostasis
Source: Cancer Res Commun. 2024 Sep 10;4(9):2374–83. doi: 10.1158/2767-9764.CRC-24-0101 (PMC11384508; doi:10.1158/2767-9764.CRC-24-0101)
Supplement: Supplementary Figure S2 — Blocking Netrin does not affect YAP levels or YAP target gene induction [file crc-24-0101_supplementary_figure_s2_suppsf2.pdf]

Supplementary Figure S2: Blocking Netrin does not affect YAP levels or YAP target gene induction

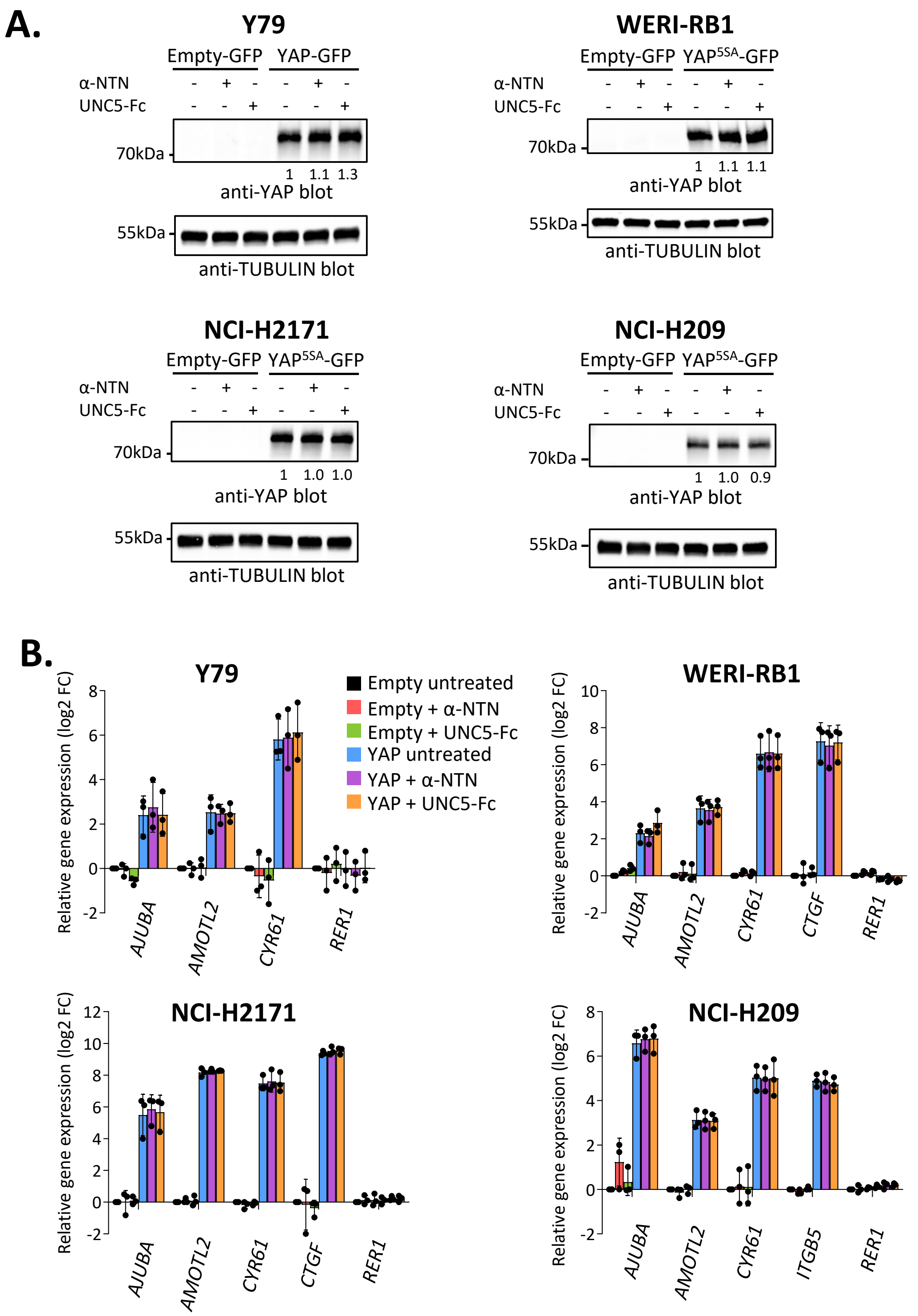

Figure S2. Blocking Netrin does not affect YAP levels or YAP target gene induction. A. YAP Western blots from Empty or YAP-expressing YAP^{off} cells untreated or treated with Netrin blocking reagents. YAP expression is quantified relative to untreated cells. n = 3. **B.** RT-qPCR for YAP target genes (*AJUBA, AMOTL2, CYR61, CTGF* and/or *ITGB5*) or a control gene (*RER1*) in Empty or YAP-expressing cells untreated or treated with Netrin blocking reagents. n = 3. Western blots (A) and RT-qPCR (B) are from the same experiments as in Fig. 4.
