# Supplementary material for: Netrin-1 and UNC5B Cooperate with Integrins to Mediate YAP-Driven Cytostasis
Source: Cancer Res Commun. 2024 Sep 10;4(9):2374–83. doi: 10.1158/2767-9764.CRC-24-0101 (PMC11384508; doi:10.1158/2767-9764.CRC-24-0101)
Supplement: Supplementary Figure S3 — Regulation of UNC5 family and NTN1 mRNA by YAP in YAP-off vs YAP-on cancers [file crc-24-0101_supplementary_figure_s3_suppsf3.pdf]

Supplementary Figure S3: Regulation of UNC5 family and NTN1 mRNA by YAP in YAP^{off} vs YAP^{on} cancers

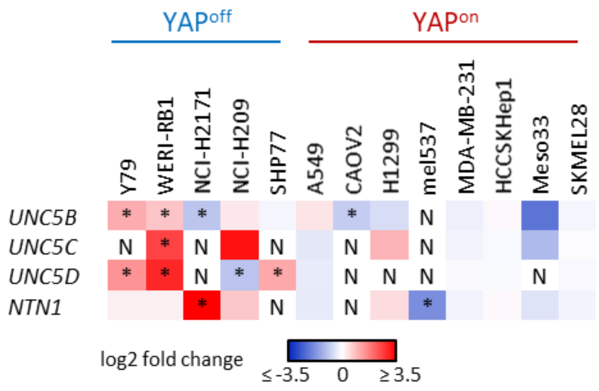

Figure S3. Regulation of UNC5 family and NTN1 mRNA by YAP in YAP^{off} vs YAP^{on} cancers. YAP induces expression of these proteins specifically in YAP^{off}, but not YAP^{on} cancers. * FDR < 0.05; N = not detected in the dataset. Data for YAP^{off} cancers is also shown in Fig 3H.
